# Degradation Performance Investigation of Hydrothermally Stressed Epoxy Micro and Nanocomposites for High Voltage Insulation

**DOI:** 10.3390/polym14061094

**Published:** 2022-03-09

**Authors:** Faiza, Abraiz Khattak, Ahmad Aziz Alahamdi, Muhammad Bilal Iqbal

**Affiliations:** 1 School of Natural Sciences, National University of Sciences and Technology (NUST), Sector H-12, Islamabad 44000, Pakistan; faizaijaz710@gmail.com; 2 Department of Chemistry, COMSATS University Islamabad, Lahore Campus Defence Road off Riwind Road, Lahore 56000, Pakistan; 3 High Voltage Laboratory, United States-Pakistan Center for Advanced Studies in Energy, National University of Sciences and Technology (NUST), Sector H-12, Islamabad 44000, Pakistan; bilaliqbalmgt@gmail.com; 4 Department of Electrical Engineering, College of Engineering, Taif University KSA, P. O. Box 11099, Taif 21944, Saudi Arabia; aziz@tu.edu.sa

**Keywords:** epoxy, hydrothermal aging, high voltage insulation, FTIR, hydrophobicity

## Abstract

Epoxy resins have demonstrated remarkable properties with potential for usage as high voltage insulators. However, a loss of these properties has been observed in high temperature and humid environments. In order to enhance the hydrothermal stability of epoxy resins, micro (15% SiO_2_) and nano (5% SiO_2_) silica-based composites of epoxy were fabricated and subjected to standard long term and short term accelerated hydrothermal conditions. To analyze the effect of these stresses, the samples were analyzed periodically through Fourier transform infrared spectroscopy (FTIR) for structure analysis; scanning electron microscopy (SEM) for surface analysis of long-term aged samples; and optical microscopy for the surface topography of short-term aged samples. The Swedish Transmission Research Institute (STRI) classification and contact angle measurement techniques were used for hydrophobicity analysis of long-term and short-term aged samples, respectively. After aging in both conditions, the nanocomposite showed better results as compared to the other samples. After 1000 h of aging, it showed HC-5 class of hydrophobicity, whereas EMC and NE degraded to the HC-6. In case of short-term aging, the contact angle decreased to the 64.15° and 75.05° from 104.15° and 114.9° for ENC and EMC, respectively. Also, in terms of structural degradation, ENC showed the highest structural stability after 1000 h of aging with the highest stable peak of aromatic ether at 1300–1500 cm^−1^. Microscopic observation through scanning electron and optical techniques also revealed superior performance of the nanocomposites.

## 1. Introduction

Epoxy based insulators have been showing noteworthy performance in electrical power systems. In many applications, epoxy has the ability to substitute ceramic insulators that are brittle in nature [1]. If electrical applications of both types are compared, epoxy material gives much better properties during usage, which makes it a first choice for modern electrical power systems [2,3,4,5]. Epoxy insulators have better resistance to mechanical stress, flashovers, anti-tracking, and erosion [6,7,8,9]. Epoxy resins also possess tremendous properties which can be used as insulators (for example, better hydrophobicity, better mechanical strength, high dielectric strength, and easy processing for usage in power systems) [10,11,12,13]. Epoxy insulators have been used recently for attaining insulating and dielectrics applications in electrical power systems [14]. It is useful insulating material due to its compact structure in comparison to ceramic insulators [15]. Major drawback of epoxy insulators is that they degrade over time under environmental stresses and their properties must be improved to have a longer service life [16]. To improve the epoxy, which has degradation problems in outdoor environments, different methods could be employed such as filler incorporation, coating with a stable material, and by making blends of different polymers [17]. Previously Messerer et al. coated zoelec ECP on the surface of the insulator to enhance the flashover voltage of the insulator [18]. Li et al. used a sputtering technique to coat the Cr_2_O_3_ on the surface of the epoxy insulator to enhance its properties [19]. Different types of fillers such as titania, silica, alumina, and zinc oxide are being used to enhance the stability of epoxy resins against external stresses [20]. Inorganic oxide nano-particles have been gaining attention due to their high thermal stability and mechanical strength. Shao et al. deposited the silica on the surface of epoxy to increase the flashover voltage [21,22]. Zhai and their team studied 2, 6, and 10 wt.% alumina-based epoxy composites and reported their UV degradation. They found that 6 wt.% composites expressed the best performance in comparison to neat samples [23]. Erosion and tracking along-with flashover performance was reported for silica-based epoxy composites for 25 kV outdoor applications. Significant enhancements in erosion were recorded and flashover did not occur even above 20% of nominal voltage [24]. Epoxy-silica nanocomposites were tested for high voltage insulation, and it was concluded that nanocomposites exhibited improved breakdown strength and dielectric response [25]. Epoxy composites have been constantly reported [26] for their improved characteristics over neat epoxy. However, some of the studies still need to be conducted before its recommendation for harsh environments. For example, as with other polymeric insulation, detailed performance analyses for high temperature/water environments are of utmost importance [27,28,29].

Keeping in view what has been mentioned above, this study presents a fabrication of both micro and nano composites, along with the neat epoxy, and a comparison of their stability against short and long-term hydrothermal stresses. The micro and nano epoxy composites are subjected to short and long term aging conditions and their behavior in terms of structural changes and superficial properties are analyzed under different intervals of aging cycles.

## 2. Materials and Methods

### 2.1. Materials

Nano silica having size of 12 nm was purchased from Degussa (Evonik) Chemical Co., Los Angeles, CA, USA, and micro silica 5 µm in size was procured from Wuhan Newreach Chemical Co., wuhan, China. epoxy (Eposchon A and B; A: Diglycidyl Ether of Bisphenol A-Primer and B: hardener Meta Phenylene) were purchased from General Electric Co., New York, NY, USA, Lanxess Co., Köln, Germany and Justus Kimiaraya, Jakarta, Indonesia.

### 2.2. Fabrication of Epoxy Composites

**Figure 1:**
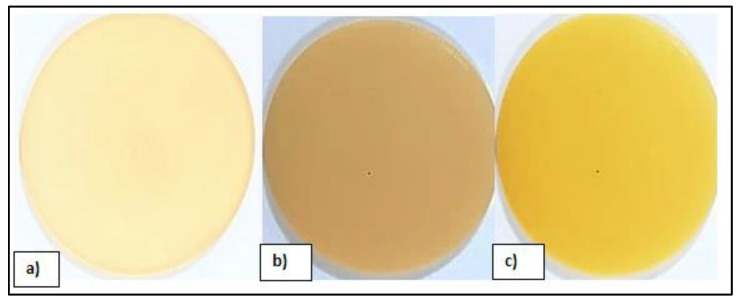
Prepared samples (**a**) neat epoxy (**b**) epoxy nanocomposite (**c**) epoxy microcomposite.

First of all, dispersion of nanosilica was achieved by using an ethanol solution by keeping it in ultrasonic bath for 90 min. After that, silane was added for surface functionalization of the filler. Surface functionalization is needed for better chemical interaction of fillers and epoxy material. In the next phase, the epoxy was added into the mixture with the help shear mixer at rotational speed of almost 3500 rpm for almost 17 min. The solution was again kept in an ultrasonic bath to make it more dispersed and to give it a better interactive structure. After that, the mixture was kept at temperature higher than the boiling temperature of ethanol in vacuum to evaporate it completely. Finally, a hardener was added and mixed in this solution. Then, the bubbles were removed from the solution by setting aside in vacuum at 27 mmHg. At the end, the mixture was poured into molds to attain the required dimensions. Prepared samples were circular in shape having 80 mm diameter and 3 mm thickness (as shown in Figure 1).

### 2.3. Experimental Setup for Degradation

The prepared samples were subjected to hydrothermal aging, and two different types of setups were used for this purpose (one for short term and another for long term aging, in order to check the hydrothermal effect on prepared samples). 

#### 2.3.1. Short Term Aging Setup

**Figure 2:**
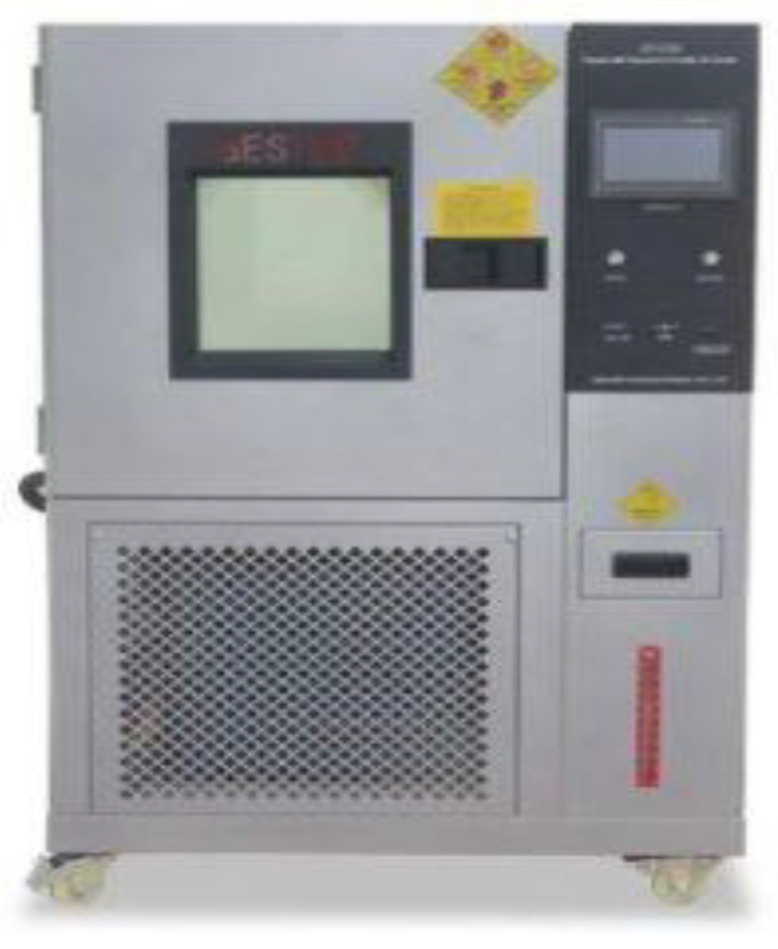
Short-term hydrothermal aging setup.

An automated environmental chamber by UTSTESTER with controlled temperature and humidity was employed shown in Figure 2. The samples were subjected to the chamber for 60 h at 70 °C with 80% humidity content. The effect of hydrothermal stresses was checked after every thirty hours. In this way, two cycles were conducted and back bone analysis was carried out after each cycle using FTIR analysis.

#### 2.3.2. Long Term Aging Setup

**Figure 3:**
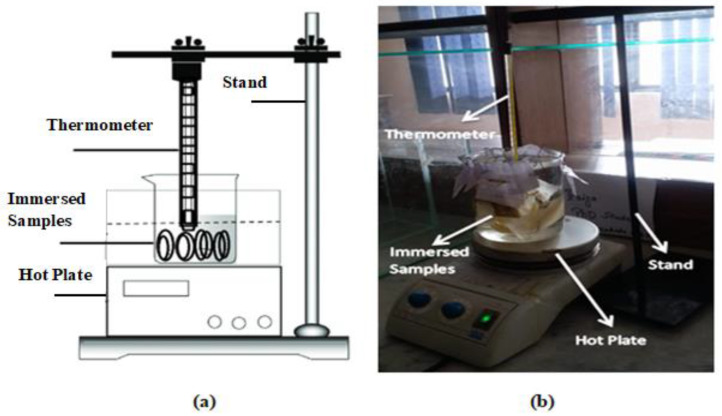
Long-term aging setup (**a**) and schematic diagram (**b**).

Experimental setup for long term aging study was developed by using a glass beaker in which samples were immersed in water and subjected to accelerated heating at 65 °C to 70 °C. The experiment was designed for 1000 h, and five cycles were carried out. In this way, to check the degradation after each cycle, analysis techniques were employed after every 200 h. A schematic diagram and image of the experimental setup are given in Figure 3.

### 2.4. Analysis Techniques

#### 2.4.1. Swedish Transmission Research Institute (STRI) Classification Method

**Figure 4:**
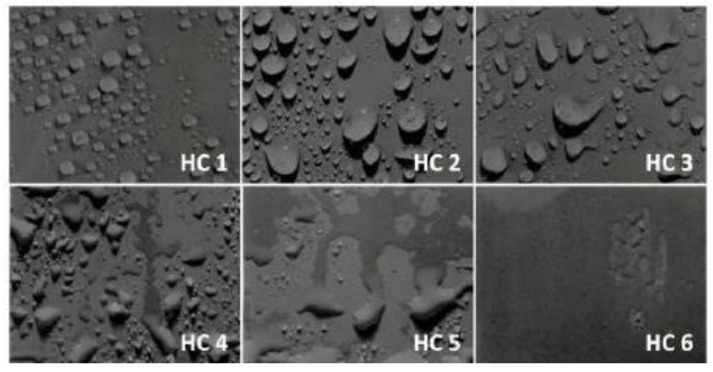
STRI hydrophobicity guide.

Swedish Transmission Research Institute (STRI) classification was employed to check the hydrophobic behavior of the samples during long term aging. Water was sprayed on to the samples and high-resolution images were taken after 20 min and compared with the standard STRI classification (as given in Figure 4).

#### 2.4.2. Contact Angle Measurements

Contact angle measurements were employed to check the constant minor changes in hydrophobicity during short term aging. For this purpose, VCA OPTIMA from ASTP (Stationsweg 28A, 2312 AV Leiden, The Netherlands) was used which consist of syringe filled with distilled water having droplet of 0.5 μL. As during short term aging, a very smaller change in water angle takes place. So as to measure the smaller changes, contact angle measurement was employed for the short-term aging experiment.

#### 2.4.3. Optical Microscopy

Optical microscopy was used at a 100 µm resolution and 20× magnification. It is evident from the literature that in short-term aging, only surface roughness takes place with prolonged aging conditions, crosslinking, and chain scission [30]. So, optical microscopy was used to find out changes or roughness on the surface during short term aging. 

#### 2.4.4. Scanning Electron Microscopy (SEM)

To analyze the surface morphology for long-term aging, a field emission scanning electron microscopy (FESEM model MIRA3 TESCAN Zeiss Supra 55 VP, Jena, Germany) was employed. Carbon coated samples were placed on stubs and images were taken. As long-term aging might affect morphology of the specimen, it is important to use sensitive technique. Thus, SEM was employed for in depth analysis.

#### 2.4.5. Fourier Transform Infrared Spectroscopy (FTIR)

FTIR analysis was performed after both aging methods by using Bruker platinum ATR model Alpha, Ettlingen, Germany with a spectral range of 4000–500 cm^−1^. Absorbance values were obtained against wave number (cm^−1^) by placing the sample directly on a diamond scanner.

## 3. Results and Discussion

### 3.1. Hydrophobicity Analysis

#### 3.1.1. Contact Angle Measurement

**Figure 5:**
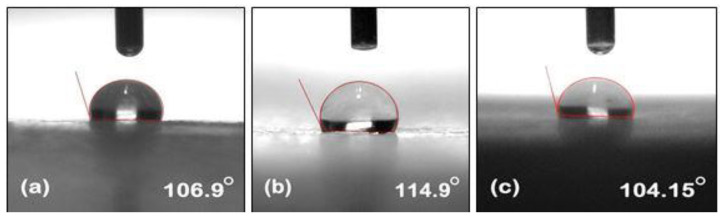
Measurement of contact angle of Unaged (**a**) neat epoxy (**b**) epoxy microcomposite (**c**) epoxy nanocomposite.

Contact angle measurements were carried out by using the contact angle meter. The contact angle images of unaged NE, EMC, and ENC could be seen in Figure 5. A higher value of the contact angle refers to the more hydrophobic nature of the material. Pure epoxy shows angle of 104° and many studies have been reported which shows that its value decreases with aging. The same trend was seen in our study. However, filler incorporation showed resistance towards a loss of hydrophobicity [31]. The contact angle of neat epoxy before aging was 106.9°, and it reduced to 64.15° and 51° after 30 and 60 h of aging, respectively. Before aging, EMC and ENC showed angles of 114.9° and 104.15°, respectively and reduced to 71.1° and 87.3° after 30 h of aging. A further decrease to 64.15° and 75.05° was seen after 60 h of aging. It could be seen that there was not much difference initially in the contact angles of all of the samples, which could be furthered verified from the STRI hydrophobicity classes where NE and EMC showed HC-2 and ENC showed HC-1 class of hydrophobicity. After aging, the decrease in contact angle can be seen but EMC and ENC showed resistance towards loss of hydrophobicity.

**Figure 6:**
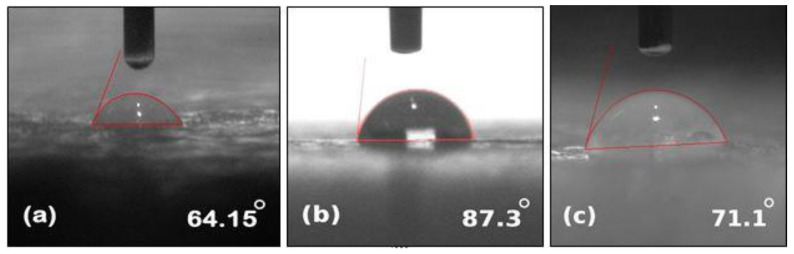
Measurement of contact angle of 30 h aged (**a**) neat epoxy (**b**) epoxy microcomposite, and (**c**) epoxy nanocomposite.

**Figure 7:**
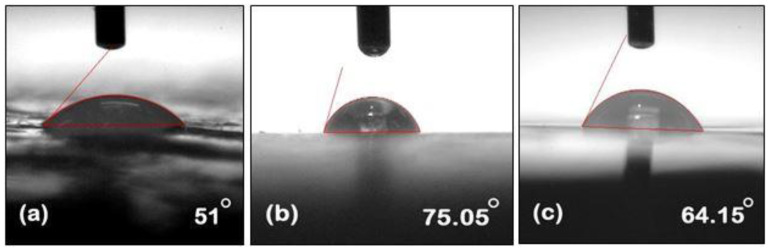
Measurement of contact angle of 60 h aged (**a**) neat epoxy (**b**) epoxy microcomposite, and (**c**) epoxy nanocomposite.

The degradation in the contact angle occurred due to hydrolysis of different functional groups in epoxy matrix (further discussed in the FTIR section). It is evident from Figure 6 and Figure 7 that incorporation of filler into the polymer matrix reduces the extent of loss of hydrophobic behavior and enhances its stability against hydrothermal stresses. As nano and micro silica provide a higher surface area, a high temperature bearing capacity and interaction with epoxy makes it more stable.

Nanosilica epoxy composite showed the least degradation in terms of contact angle. This may be due to the fact that the surface area of nanosilica is greater and shows better interaction with epoxy networkmakes it more stable against aging conditions.

#### 3.1.2. STRI Analysis

Hydrophobic character of all of the prepared samples were analyzed by their comparison with HC-1 to HC-7 classes of Swedish Transmission Research Institute (STRI) hydrophobicity classification standard given in Figure 4, where HC-1 corresponds to most hydrophobic and HC-7 is the most hydrophilic class. Variation in hydrophobic character was observed by changing filler concentration in polymer matrix. Hydrophobic images of all of the samples were taken after every 200 h. A total of five cycles were performed and final images were taken after 1000 h. 

Loss of hydrophobic behavior after applying hydrothermal stress can be seen clearly in all samples. Hydrophobicity class HC-2 was observed in case of unaged neat epoxy and silica micro composite, while the nanosilica composite showed hydrophobicity class HC-1 After first cycle of 200 h, neat epoxy and EMC retained the same class of hydrophobicity. After that, continuous loss of hydrophobic behavior was seen after every 200 h in the case of the neat epoxy. HC-3 and HC-4 class was seen after the third and fourth cycle and finally degraded to HC-6 after the fifth cycle. 

On the other hand, silica micro and nano composites showed better stability against their loss of hydrophobic behavior. Silica micro composite showed degradation after the second cycle and HC-3 class was seen. After that, a constant loss of hydrophobicity was seen and HC-4, HC-5, HC-6 classes were observed after the third, fourth, and fifth cycles, respectively. In the case of the silica nanocomposite, a continuous loss of hydrophobicity from HC-1 to HC-2 was observed, which was maintained until the second cycle and then further degraded to HC-3 and HC-5 after the third and fourth cycles, respectively, (being retained even after the last cycle).

These results showed that hydrophobicity decreased due to the deposition of hydroxyl groups on the surface of the epoxy. These groups attract water molecules that lead to a decrease of hydrophobicity. Loss of hydrophobicity at early stages of aging in neat epoxy occurred due to the roughness in surface of epoxy [32]. Loss in hydrophobicity may also occur due to the hydrolysis of ester groups. These processes cause the degradation of the epoxy surface and destabilized the chemical bonding, which contributed to its hydrophilic nature [33]. 

The micro silica composite showed better stability of its hydrophobic behavior in comparison to the neat epoxy, but more degradation was observed in comparison with the silica nano composite.

The nano silica composite showed better retention of hydrophobicity as compared to neat epoxy and silica micro composites. By the addition of fillers, better results were obtained due to the better surface area provided by the filler. An increase in surface area from micro to nano filler water repellency also occurred. 

### 3.2. Structural Analysis

#### 3.2.1. Optical Microscopy

**Figure 8:**
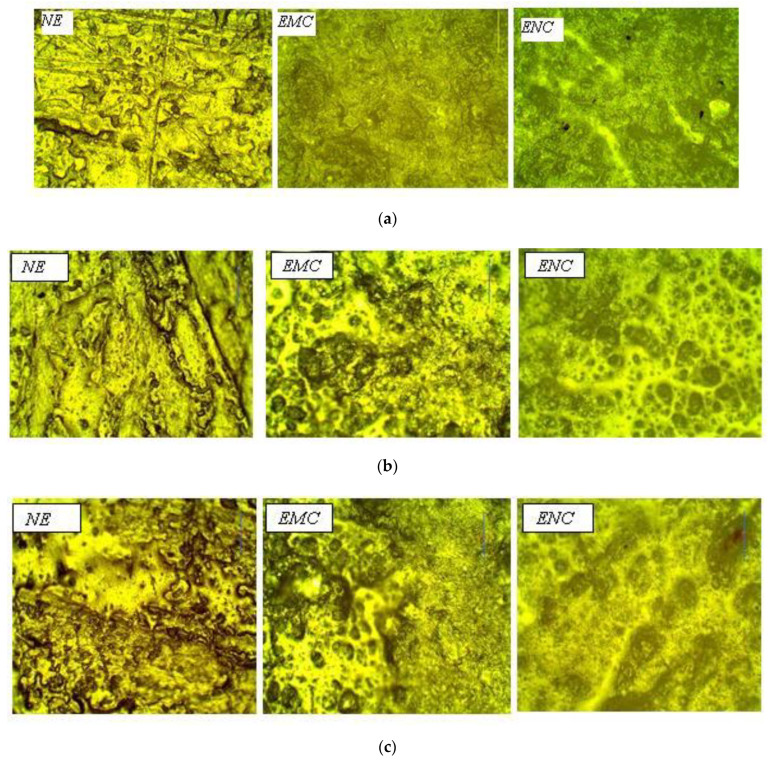
(**a**) Optical microscopy images of unaged samples. (**b**) Optical microscopy images of 30 hrs aged samples. (**c**) Optical microscopy images of 60 h aged samples.

Optical microscopy was employed to analyze the surface roughness during short-term aging. An unaged sample can be seen in Figure 8a. The surface of EMC and ENC was smoother than NE, which is due to the crystalline nature of the filler. It was observed that surface roughness and voids appear after applying hydrothermal stresses. The extent of degradation was highest in the case of neat epoxy and lowest in the case of ENC. 

These voids and roughness appeared due to the oxidation on surface. The degradation was minimized in case of micro composite due to higher surface area of micro and nano silica, which reduced the oxidation at the surface. The silica filler is stable under higher temperatures and forms a strong interaction with the polymer matrix, resulting in increased resistance towards oxidation. It could be seen from Figure 8b,c that minimum voids were created on the nanosilica-based epoxy composite. However, in the case of the microcomposite, the voids were greater than the nanocomposites but less than the neat epoxy.

Analysis of optical microscopy results showed that the nano silica-based composite was more stable and retained its surface against elevated temperature and humidity. 

#### 3.2.2. Scanning Electron Microscopy (SEM)

**Figure 9:**
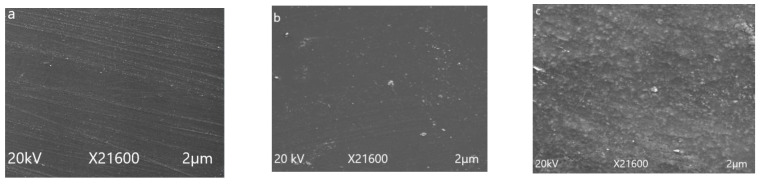
Scanning electron microscopy images of unaged samples. (**a**) neat epoxy, (**b**) EMC, (**c**) ENC.

Scanning electron microscopy (SEM) was used to analyze the surface morphology of epoxy material and its degradation after applying long term hydrothermal stresses. SEM images of all of the prepared samples were taken before and after 1000 h of aging at 100 µm. SEM images of un-aged samples are given in Figure 9. Smooth surface and fine dispersion of micro and nano fillers could be seen clearly from the images. Before applying stresses, no cracks were seen at surface of the virgin, micro-, and nanocomposites. 

**Figure 10:**
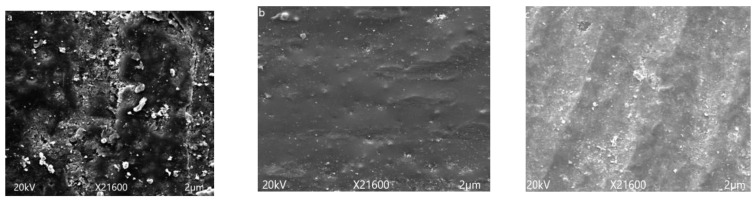
Scanning electron microscopy images of 1000 h aged samples. (**a**) neat epoxy, (**b**) EMC, (**c**) EnC.

In Figure 10, SEM micrographs of aged samples after five cycles are given. After 1000 h of aging distortion in surface smoothness, cracks and holes are visible in the images. However, the extent of degradation varies with the amount and size of silica added to the epoxy matrix. Maximum distortion of surface smoothness and cracks were seen in the case of the neat epoxy. The 15% micro silica composite showed cracks and holes on the surface, but this distortion was very much less as compare to the neat epoxy. In the case of the 5% nano silica composite, no holes, cracks and degradation were observed. This improvement in structural stability by the addition of fillers is due to the better intactness of filler with the polymer and nano composite, providing more surface area and better intactness with the polymer matrix as compared to the micro filler. These results also support the hydrophobicity results and images given and discussed earlier.

### 3.3. Fourier Transform Infrared Spectroscopy (FTIR)

FTIR was employed to study the variation in absorbance of different functional groups in the epoxy, which indicated the extent of degradation in its structure, as the increase or decrease in intensities of the absorbance band corresponds to the degradation and interactions involved during hydrothermal aging. Table 1 shows the specific assigned functional groups of epoxy backbone.

**Figure 11:**
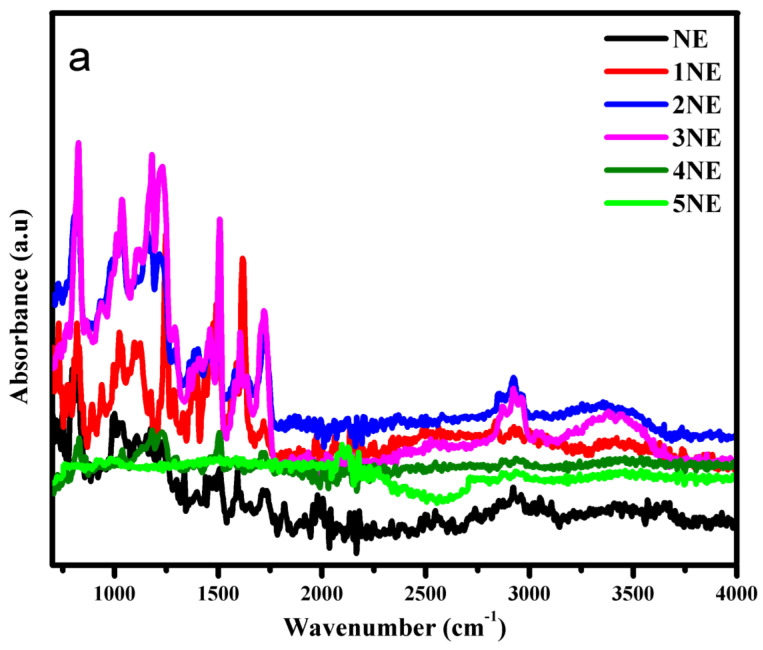

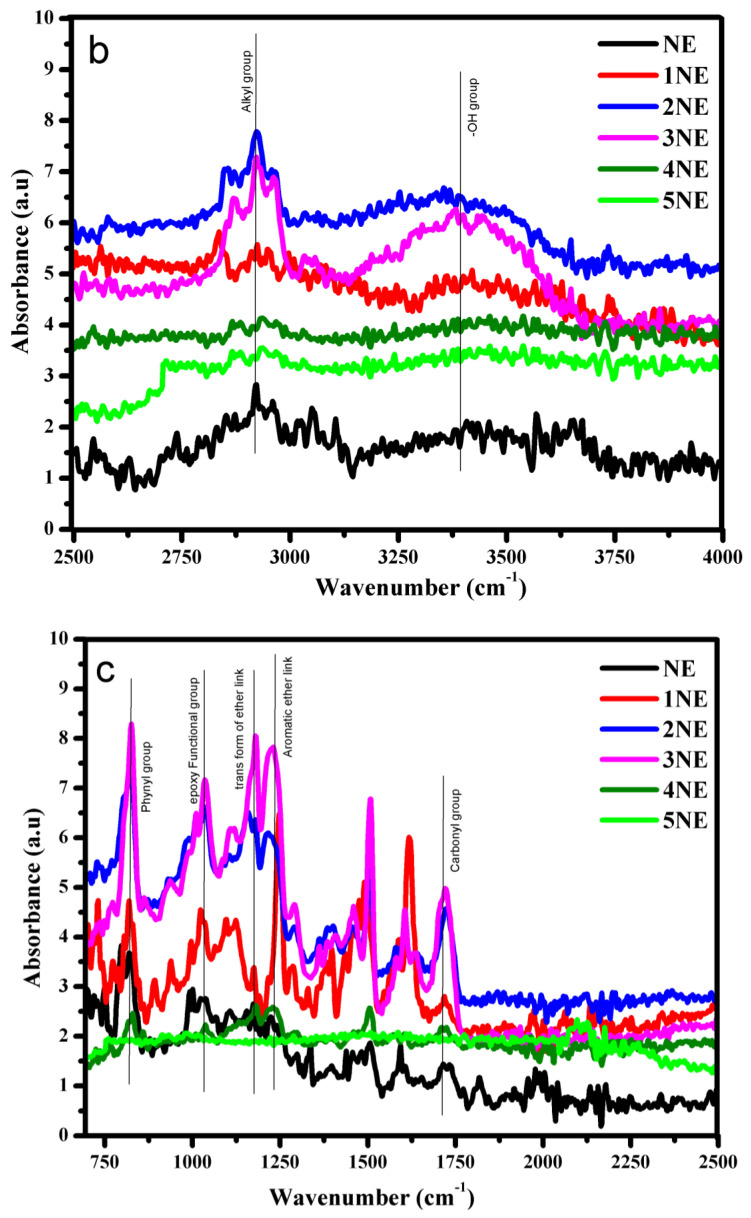
FTIR analysis of neat epoxy in wavelength range (**a**) 750–4000 cm^−1^ (**b**) 2500–400 cm^−1^ (**c**) 750–2500 cm^−1^.

**Figure 12:**
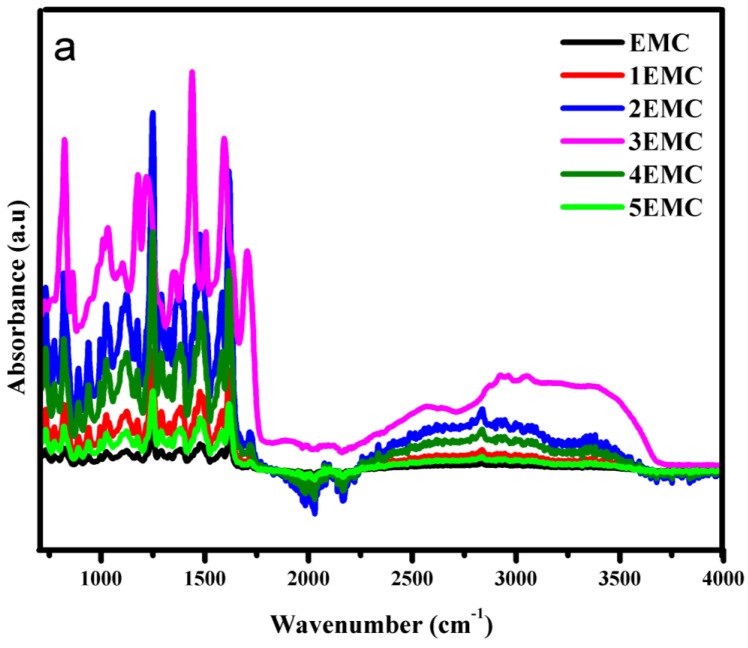

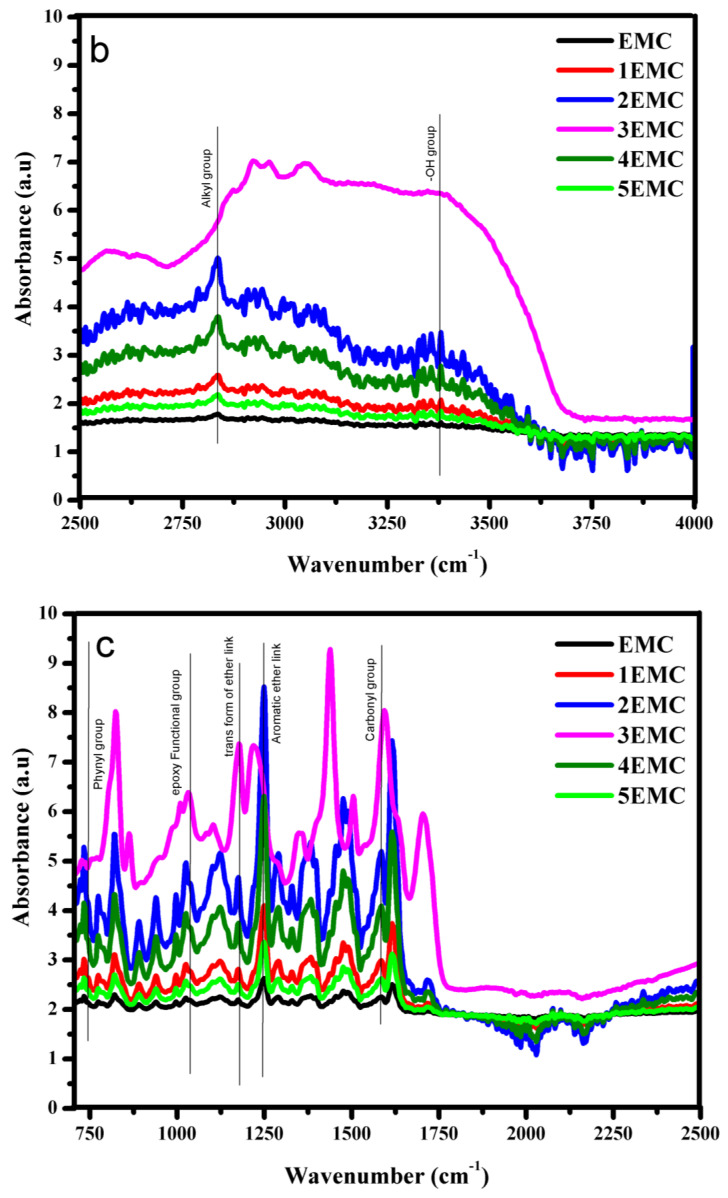
FTIR analysis epoxy microcomposite in wavelength range (**a**) 750–4000 cm^−1^ (**b**) 2500–400 cm^−1^ (**c**) 750–2500 cm^−1^.

Long-term aging results of FTIR after each cycle were recorded. The band between “2700 to 3500 cm^−1^” corresponds to the –OH group. It is evident that the NE intensity of this band gradually increased up to the second cycle. Then, the loss of the –OH group can be seen during the third, fourth, and up to the fifth cycles. This is due to the diffusion of water into epoxy chain. After 400 h, a loss of water can be seen due to degradation in the epoxy backbone [33]. Trends for neat epoxy functional groups can be seen in Figure 11a–c. In case of EMC, an increase in the intensity of the –OH band can be observed up to the third cycle (600 h), and its loss in the fourth and fifth cycles can be seen in Figure 12b. However, ENC showed water sorption up to the fourth cycle (800 h), and finally a loss of water was observed in the last cycle. 

**Figure 13:**
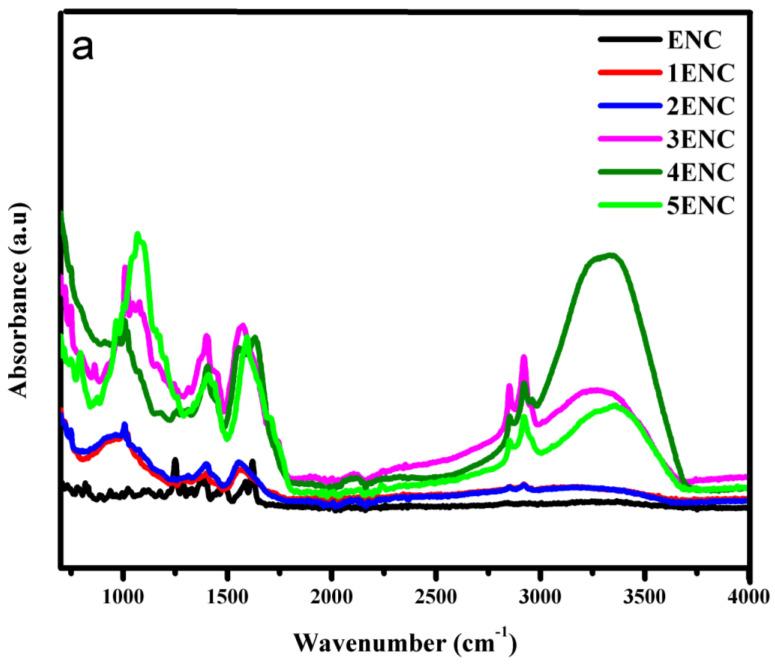

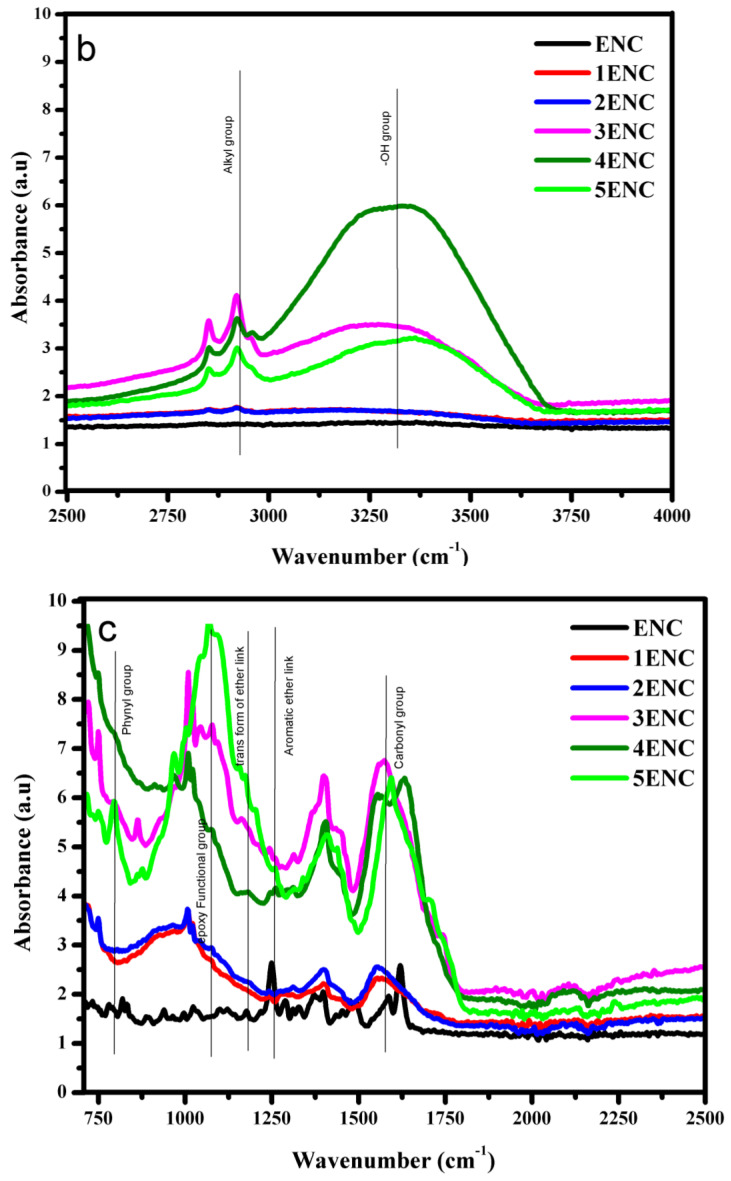
FTIR analysis of epoxy nanocomposite in wavelength range (**a**) 750–4000 cm^−1^ (**b**) 2500–400 cm^−1^ (**c**) 750–2500 cm^−1^.

Absorbance intensities of alkyl group can be seen between bands 2750–3000 cm^−1^ in Figure 11b, Figure 12b and Figure 13b.This phenomenon indicates that prolonged hydrothermal aging causes irreversible degradation in the epoxy chain. It is evident that due to water sorption, ENC showed least degradation. In the case of NE, it was observed that the intensity of alkyl group increased up to second cycle (400 h) and showed degradation during the first and second cycle. After 400 h, maximum hydrolysis was observed as the alkyl group peak was 267% of the virgin sample. After that, a decrease in alkyl group can be seen due to further oxidation. A decrease in intensities was seen during the third, fourth, and fifth cycles. in In the case of the EMC, an increase in intensities was recorded up to the third cycle (which was 298%) and then a decrease in fourth and fifth cycles was seen. However, in case of ENC the increase in intensity was up to the fourth cycle (800 h) that was 292%. Then a decrease in the final cycle was observed. Same trend was seen for carbonyl (1600–1750 cm^−1^) and p-Phenyl (700–800 cm^−1^) groups. Finally, after the 1000 h of aging the alkyl peak was 128%, 116% and 198% of the virgin peaks for NE, EMC, and ENC respectively. The degradation epoxy back bone can be divided into two steps. The first step involves a breakdown of groups, which resulted in increase in absorbance intensities of these groups and second step involves oxidation which causes decrease in absorbance intensities and leads to the complete degradation of the epoxy.

The increase in intensities of the alkyl, p-Phenyl, and carbonyl groups was due to the hydrolysis of the ester linkages, which resulted in increase in intensity Of –OH band. After that, the decrease might be due to the oxidation of this group, which shows the degradation in epoxy backbone [33]. The absorbed water can attack chain of epoxy resins, which can result in leaching and chain breakdown by distortion of crosslinking. An increase in ether peaks up to the second, third, and fourth cycles for NE, EMC, and ENC, respectively, can be seen clearly in Figure 11c, Figure 12c and Figure 13c. The decrease in intensities was due to the further oxidation of the hydrocarbon backbone. This degradation resulted into the increased intensities of C=C and C=O. The maximum absorbance intensities for carbonyl groups were 333%, 285%, and 261% of the virgin samples for NE, EMC, and ENC respectively. After that further degradation was recorded. However, the intensity remained almost same for ENC till 1000 h and no further degradation was recorded.

For quantitative analysis relative absorbance intensities were measured. As the aromatic ether bond at 1300–1500 cm^−1^ is the most stable bond of epoxy and it does not react with water and oxygen easily. After 1000 h of aging the intensity of aromatic ether group was 80%, 166 %, and 170% of the original peak. So, the relative absorbance intensities were calculated for this functionality by using following equation.

$$Ir=\frac{Iepoxy}{Ireference}$$

where, *Ir* is the relative intensity.

**Figure 14:**
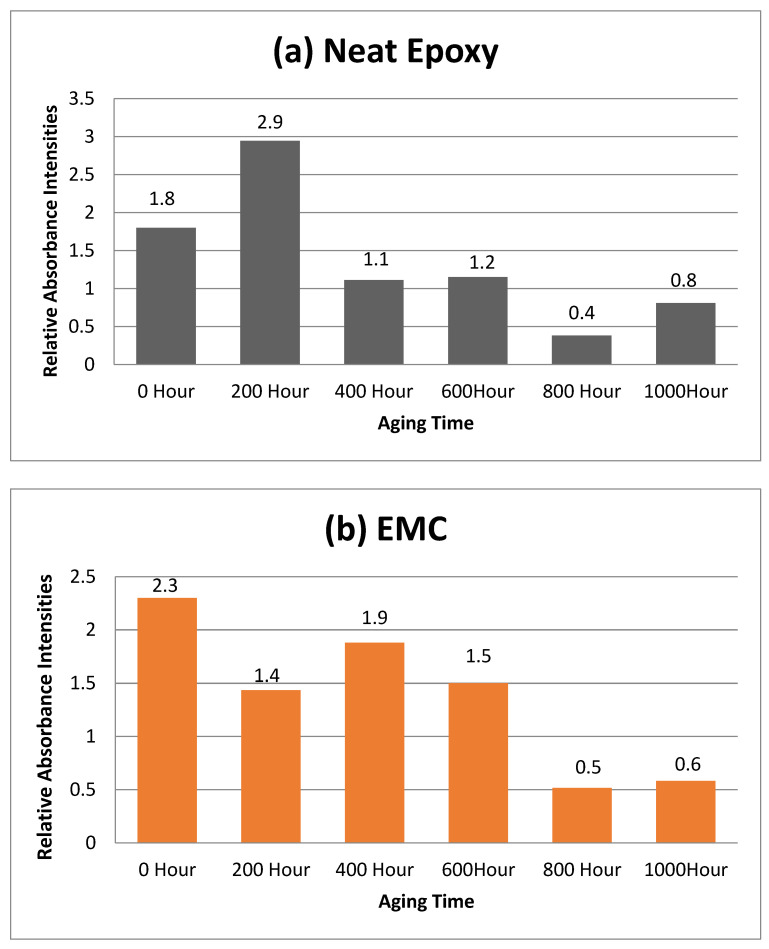

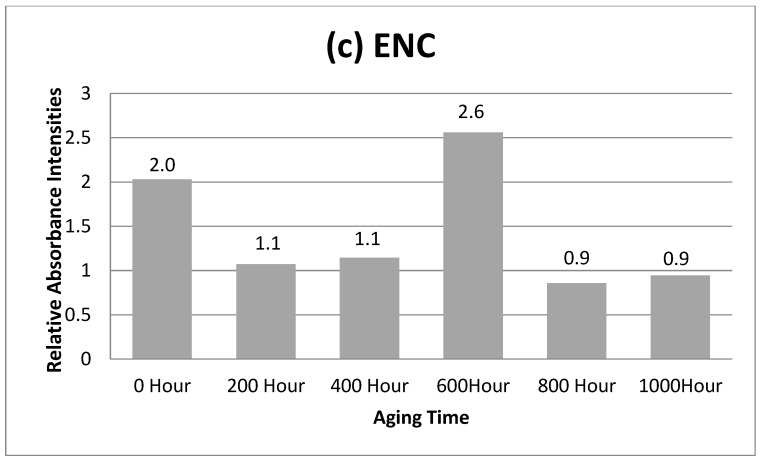
Changes of relative absorbance intensities of epoxy functional groups with long term hydrothermal aging.

The comparison of relative absorbance intensities are given in Figure 14. The neat epoxy drastically increased absorbance from 1.8 to 2.9 after 200 h, which may be due to the decrease in crosslinking of the chains. After that, a decrease from 2.9 to 1.1 in intensity of the absorbance intensity was noticed after 600 h, which is due to the rapid cure reaction of the epoxy. Almost same the intensity was retained after 800 h, and a further decrease to 0.4 was seen after 1000 h of aging. In the case of EMC, after 400 h a decrease in intensity could be seen due to cure reaction and after 600 h an increase in intensity from 1.4 to 19 could be seen. After that, continuous decrease in the intensities can be seen up to 100. However, in the case of ENC a continuous decrease in intensities could be seen.

An increase at 600 h could be seen. It is evident that degradation in crosslinking occurred was after the second, third, and fourth cycles for NE, EMC, and ENC, respectively. A statistical tool was used to analyze the data of Figure 14a–c. For this purpose, a paired t test was employed to check the difference between unaged and aged samples. However, before applying the t test, the normality of the data was checked by using the Shapiro–Wilk test as degree of freedom is small. It is evident from the Table 2 that all data points are normally distributed as *p* > 0.05.

In the next step, samples were analyzed using one way ANOVA, which was employed to check the relative standard deviation and standard error in the absorbance values of the samples. From Table 3 and Table 4, it can be seen that nanocomposite showed least deviation as well as least std error in its absorbances.

The *p*-value between the groups is *p* > 0.05, which suggests that the difference between all three samples are not significant. However the std deviation value of absorbance is lowest in case of ENC which represents its slow aging.

FTIR analysis for short term aging was also carried out to check the degradation in structure of epoxy backbone.

**Figure 15:**
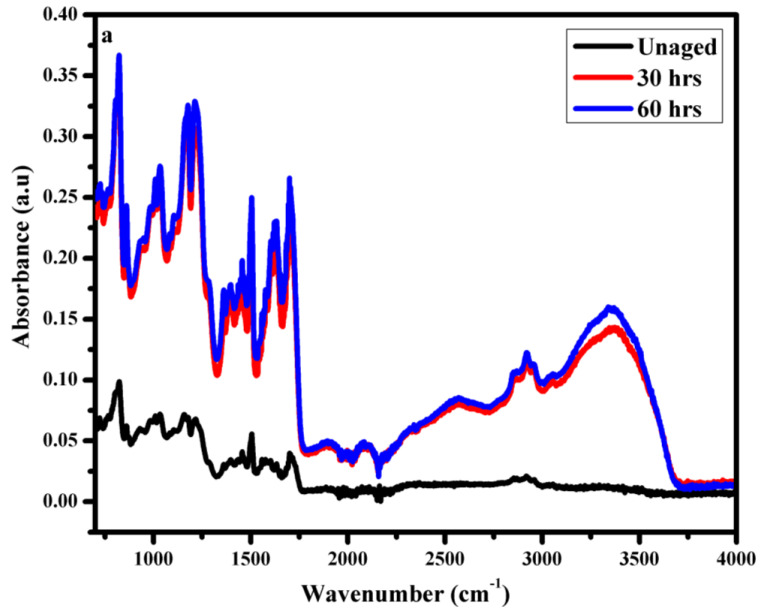

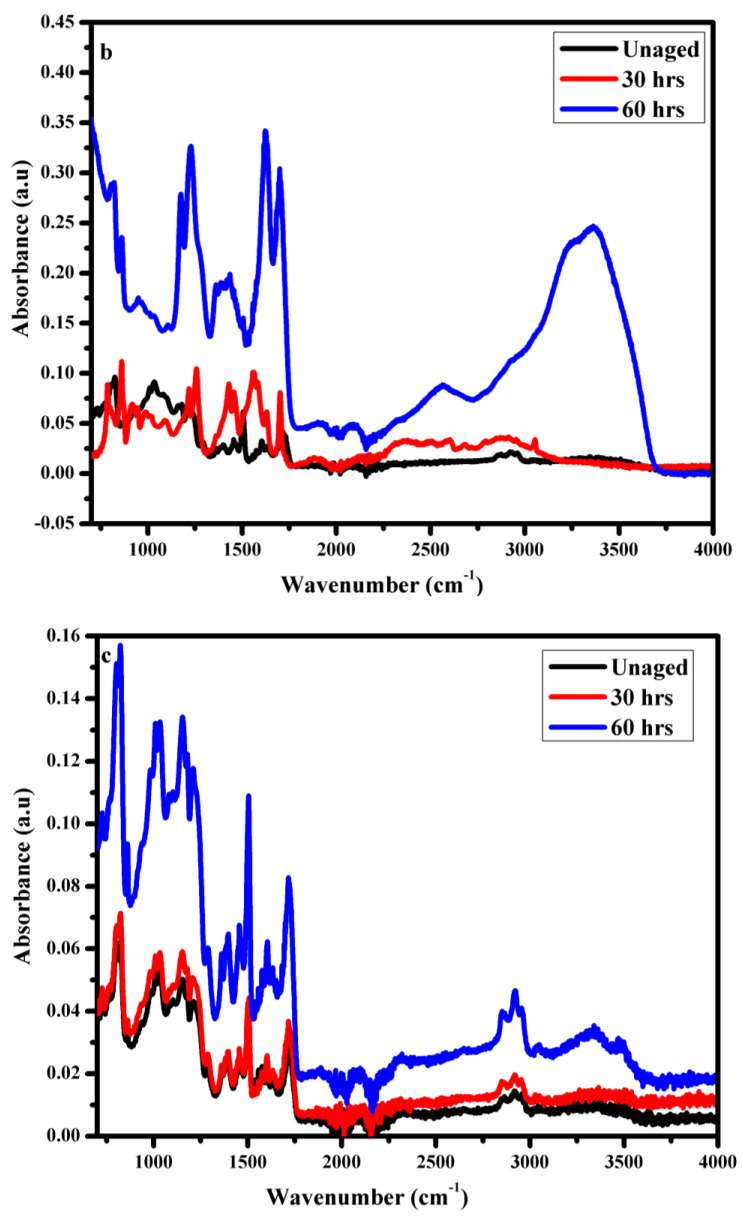
FTIR analysis of short-term aging (**a**) neat epoxy (**b**) epoxy microcomposite, and (**c**) epoxy nanocomposite.

It could be seen in Figure 15a–c that in case of short-term, aging there was not much difference in the absorbance of the samples. This indicates only surface degradation and oxidation. However, it is evident that water sorption in the case of the neat epoxy after 30 h of aging was observed, but no –OH band from 3000–3500 was observed in case of EMC and ENC. This is due to the more stable structure of nano and micro composite. Similarly, the increase in absorbance of alkyl group at 2750–3000 cm^−1^ due to hydrolysis was prominent in the neat epoxy, and there was no significant increase in the band intensity in EMC and ENC after 30 h. A small increase can be seen after 60 h. However, the increase was higher for EMC. This indicates the more stable structure of ENC. The same trend was recorded for all of the other bands.

## 4. Conclusions

An epoxy neat sample (NE), epoxy 5% nano silica composite (ENC), and epoxy 15% silica micro-composite (EMC) were prepared and subjected to short term as well as long term aging using different accelerated experimental setups of hydrothermal stresses. From the FTIR results, an increase in peak of carbonyl (C=O) was recorded, showing the oxidation of the backbone. For long-term aging, detailed degradation analysis was carried out and it could be seen that maximum oxidation in terms of an increase in intensity of carbonyl group was observed. In NE, 333%, 285% in case of EMC, and 261% was recorded for ENC after t the third cycle. Also, the most stable group (which is the aromatic ether group in ENC) showed maximum stability with 170% peak intensity of virgin sample as compared to 80% and 166% for NE and EMC, respectively. Similarly for the alkyl group, after the 1000 h of aging the peaks were 128%, 116%, and 198% of the virgin peaks for NE, EMC, and ENC, respectively. Hydrophobicity classification was carried out using contact angle measurements for short-term aging. The ENC showed higher resistance towards loss of hydrophobicity. The contact angle for NE was 106.9° and was decreased to the 51° after 60 h of aging. However, EMC and ENC showed contact angle of 114.9° and 104.15 which decreased to 75.05 and 64.15, respectively after 60 h of aging. Similarly in case of long-term aging initially NE and EMC shows HC-2 and ENC shows HC-1 class of hydrophobicity and after 1000 h of aging EMC and NE shows HC-6 and ENC shows HC-5 class of hydrophobicity. In both cases, the loss of hydrophobic behavior was minimum in the case of ENC and highest in the case of NE. Results from both optical and scanning electron microscopies and SEM methods revealed that the nanocomposites exhibited least surface degradation as compared to NE and EMC.

## Figures and Tables

**Table 1:** FTIR bands assigned to epoxy.

Groups Name	Wavenumber (cm^−1^)
–CH_3_ Group Asymmetrical Stretching (Methyl)	~2965
–CH_2_ Group Asymmetric Stretching	~2928
EWA-CH_2_ Group Symmetric Stretching	~2871
C=O Vibration Stretching	~1725
Aromatic Ring C=C Stretching	~1605
Aromatic Ring C-C Stretching	~1506
Stretching of Symmetrical aromatic C-O-C	~1033–1050
Deformation in Aromatic –CH bending	~829
Rocking of –CH_2_	~724

**Table 2:** Shapiro–Wilk normalization test results.

Neat Epoxy			EMC			ENC		
Stat	df	*p*	Stat	df	*p*	Stat	df	*p*
0.964	6	0.853	0.933	6	0.601	0.795	6	0.052

**Table 3:** Descriptive analysis through one way ANOVA.

		N	Mean	Std. Deviation	Std. Error	95% Confidence Interval for Mean	
						Lower Bound	Upper Bound
absorbance	NE	6	1.3067	0.96926	0.39570	0.2895	2.3238
	ENC	6	1.4333	0.70333	0.28713	0.6952	2.1714
	EMC	6	1.3667	0.70899	0.28944	0.6226	2.1107

**Table 4:** One way ANOVA analysis.

ANOVA					
Absorbance					
	Sum of Squares	df	Mean Square	F	Sig.
Between Groups	0.048	2	0.024	0.037	0.963
Within Groups	9.684	15	0.646		
Total	9.732	17			

## Data Availability

The data presented in this study are available on request from the corresponding author.
